# Advances in the Study of Exosomes as Drug Delivery Systems for Bone-Related Diseases

**DOI:** 10.3390/pharmaceutics15010220

**Published:** 2023-01-09

**Authors:** Jiawen Huang, Yang Xu, Yuxuan Wang, Zhiang Su, Tingting Li, Sisi Wu, Yuheng Mao, Shihua Zhang, Xiquan Weng, Yu Yuan

**Affiliations:** 1 Department of Exercise Biochemistry, Guangzhou Sport University, Guangzhou 510500, China; 2 School of Exercise and Health, Guangzhou Sport University, Guangzhou 510500, China; 3 School of Exercise and Health, Shanghai University of Sport, Shanghai 200438, China

**Keywords:** exosomes, drug delivery vehicles, bone-related diseases

## Abstract

Bone-related diseases are major problems and heavy burdens faced by modern society. Current clinical approaches for the treatment of these pathological conditions often lead to complications and have limited therapeutic efficacy. In this context, the development of nanotherapeutic platforms, such as extracellular vesicles, can improve the relevant therapeutic effects. In particular, exosomes are nano-sized, lipid bilayer extracellular vesicles secreted by many cells in mammals. Due to their innate capacity to transport materials—including proteins, lipids, and genes—among cells, as well as their innate attraction to target cells, they are considered to be a crucial medium for cell communication and are involved in a number of biological processes. Exosomes have been used as drug delivery vehicles in recent bone tissue engineering studies, in order to regulate bone homeostasis. However, the precise workings of the exosome regulatory network in maintaining bone homeostasis and its potential for treating bone injury remain unclear. To provide a fresh perspective for the study of exosomes in drug delivery and bone-related diseases, in this paper, we review recent studies on the roles of exosomes for drug delivery in bone homeostasis and bone-related diseases, as well as the composition and characteristics of exosomes and their regulatory roles in bone homeostasis and bone-related diseases, aiming to provide new ideas for the therapeutic application of exosomes in the treatment of bone-related diseases.

## 1. Introduction

Bone is one of the most vital organs in the body, and bone cells undergo continuous transformation throughout human life. It has been shown that the occurrence and progression of bone-related diseases are related to the balance of bone formation and bone resorption [1]. Bone-related diseases, such as osteoporosis and osteoarthritis, have become a global public health problem with the increasing aging population [2], the prevalence of obesity, and sedentary lifestyles. Treatment of bone-related diseases requires high doses of oral and intravenous infusion, and excessive drug concentrations in the blood may cause secondary adverse effects in other organs and tissues. In order to prevent reaching blood intoxication levels of various drugs, it is vital to maximize the effective drug concentration distribution at the site of bone lesions. Exosomes are extensively engaged in the regulation of bone formation and bone resorption, according to numerous recently published studies [3,4]. As a part of the drug delivery system (DDS), exosomes have been frequently used in recent years to treat bone-related diseases, due to their good biocompatibility, capacity to traverse biological barriers, and ease of surface modification [5,6]. However, it is still unclear how the exosome regulation network regulates bone homeostasis and whether it can be used to treat bone-related diseases. In this review, along with the pathophysiological process, clinical symptoms, and therapeutic effects related to exosomes, we discuss the functions of exosomes for drug delivery in bone-related diseases.

## 2. Exosome

### Origin and Composition

**Figure 1:**
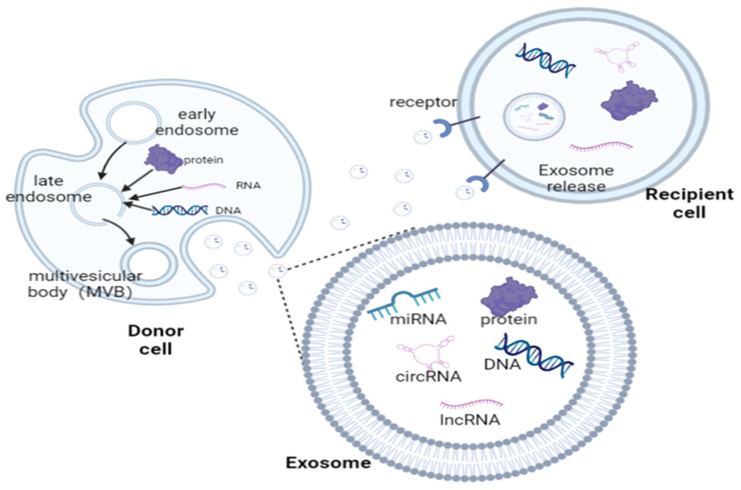
Exosome formation and their role in intercellular communication. Exosomes are formed from endosomes and serve as a link between cells, transporting various biologically active molecules to recipient cells.

Over the past decade, researchers have discovered that cells from higher animals and micro-organisms can release extracellular vesicles (EVs), ranging in size from about 40 nm to a few µm [7], including exosomes, microvesicles, and apoptotic blebs [8], and these secreted vesicles belong to a family known as extracellular vesicles. EVs are released by cells under various physiological and pathological circumstances. Exosomes are nanoscale EVs derived from the endocytic pathway—a process by which cells internalize cytoplasm, macromolecules, membranes, and receptors through an endocytic vesicle after a membrane break. Initially, exosomes were found as endocytic vesicles composed of the bilayer membrane of protocells, discovered by Harding et al. [9] and Pan et al. [10]. They have also been found in various body fluids, such as plasma [11], urine [12], cerebrospinal fluid [13], saliva, and amniotic fluid [14], in follow-up studies. Of course, the potential of plant-derived nanovesicles in human health should not be under-estimated [15,16,17]. Vesicles can carry lipids, proteins, and nucleic acids from donor cells to recipient cells, protect their cargo contents from enzymatic degradation by the internal environment, and promote their intercellular absorption [18], thus realizing material and information exchange between cells [19]. The exosomal membrane is mainly composed of lipids and proteins, including heat-shock proteins (e.g., Hsc70, Hsp70, and Hsp90), membrane transport and fusion proteins (e.g., GTPase, annexin) [20], and proteins involved in multi-vesicle biosynthesis, which enables exosomes to specifically fuse with recipient cells and release their contents [21]. The release of exosomes into the cell requires the following steps: first, endocytic vesicles fuse with early endosomes and initiate cargo sorting. Subsequently, early endosomes develop into late endosomes, which finally rejoin lysosomes. Multivesicular bodies (MVBs), which are produced by some endosomes during the maturation process and are characterized by intraluminal vesicles (ILVs), fuse with the plasma membrane and release ILVs into the extracellular environment to form exosomes [22]. The mechanisms involved in the uptake of exosomes and how their contents enter target cells remain unclear, but what is certain is that the process involves three steps, which are: (1) EVs dock with the recipient cell through specific molecular interactions or non-specific cytokinesis. (2) Upon entry into the recipient cell, the EVs target the nuclear endosome. (3) The internalized EVs release their contents via an endosomal escape pathway. Exosomes range in size from 40 to 150 nm, and their widespread presence in bodily fluids demonstrates their stability in the extracellular environment and exhibits their potential for cell–cell communications [23] (Figure 1). As a result, exosomes are currently key delivery tools for diagnosing and curing tumors and other related diseases in clinical application.

## 3. Exosomes and Drug Delivery Systems

### 3.1. Advantages of Exosomes as Drug Delivery Vehicles

DDS have evolved in recent years. Liposome- and polymer-based systems have both received extensive study; however, general nanocarriers have limited targeting, poor stability, and are easily phagocytosed by macrophages of the reticuloendothelial system in the liver and spleen [24,25]. In comparison, exosomes are derived from the nanoscale vesicle-like structures of cells with a natural biological origin and complexity. Compared with other systems, exosomes are considered one of the most promising drug delivery carriers, due to their selectivity, safety, stability, and long-distance transport of substances. Exosomes are released into the extracellular space by fusing directly with the recipient cell’s plasma membrane and transferred to the target cell by endocytosis and receptor–ligand interactions, delivering the proteins and genetic information contained therein to the target cell, along with bioactive substances [26]. These endogenous drug delivery vehicles are more adaptable to the complex environment in vivo and can successfully deliver drugs to target tissues. While improving drug efficacy, they exhibit low toxicity, low immunogenicity [27], and good biocompatibility [28], opening a new pathway for the transport of genetic and antitumor drugs.

### 3.2. Exosomes from Different Cells

Exosomes can be produced by almost all cells; however, not all of them can be used to carry drugs. There are strict screening requirements for exosomes that can be employed for drug administration, including surface protein, size, yield, and luminal composition. Exosomes derived from different cells can accurately target illnesses with different drugs to provide specific therapeutic effects. Mesenchymal stem cells (MSCs), immune cells, tumor cells, and certain cell lines are among the most prevalent cell sources. The choice of cell type from which the exosomes are produced is crucial in practical applications, as exosomes inherit numerous traits from their parental cells.

#### 3.2.1. Mesenchymal Stem Cell and Bone Tissue Cells

MSCs are pluripotent stem cells mainly found in bone marrow, adipose tissue, umbilical cord, and other tissues, and are the only cell type that can sustain extensive exosome synthesis [29]. Exosomes derived from MSCs have the capacity to enhance tissue healing, control immunological response, promote cell proliferation, and enhance drug delivery. In comparison to parental cells, they exhibit minimal immunogenicity and low carcinogenic risk [30]. Exosomes derived from bone marrow mesenchymal stem cells (BMSCs) have been discovered to be able to integrate into osteoblasts and boost the expression of osteogenic genes by Qin et al. [31]. According to another study [32], exosomes produced from MSCs could directly and rapidly communicate with chondrocytes through endocytosis, and enhanced the migration of endogenous chondrocytes to defects in vitro and in vivo. Exosomes from human umbilical cord mesenchymal stem cells (UMSCs) have also been shown to successfully block the death of BMSCs in rats with disuse osteoporosis and to prevent osteoporosis through the miR-1263/Mob1/Hippo signaling pathway [33]. 

It has been established that the transfer of active molecules from cellular sources is essential for bone homeostasis and osteocyte function. Previous studies have revealed a close connection between exosomes and osteocytes [34,35], while recent studies have shown that nearly all osteocytes are able to produce exosomes and have a regulatory function in bone remodeling [36]. The osteogenic process relies on the delivery of exosomes, which are secreted by osteoblast precursors to promote osteogenesis before differentiation into osteoblasts [37]. It has been shown [38] that myostatin-treated osteocyte exosomes drastically down-regulated miR-218 expression. During osteoblast differentiation, miR-218 is induced, which acts to promote the differentiation of undifferentiated bone marrow cells. After being endocytosed by osteoblasts, Runx2 expression may be reduced by the Wnt signaling pathway, which prevents miR-218 from growing. However, there was no obvious change in osteoclast activity after endocytosing these exosomes, indicating that miR-218 may target osteoblasts, rather than osteoclasts, in order to take part in muscle–skeletal communication, or functions as a bone potential therapeutic target for osteoporosis.

It has been found that exosomes are widely involved in the interaction between bone-forming osteoblasts and bone-resorbing osteoclasts. The coordination of these two cells to maintain bone homeostasis under various circumstances can be demonstrated in terms of the exosomal microRNAs (miRNAs) generated by osteoblasts and osteoclasts. When osteoblasts deliver RANKL-containing exosomes to osteoclast precursors, RANKL–RANK signaling is stimulated, promoting the development of osteoclasts. This osteoblast–osteoclast intercellular communication mediated by exosomes may constitute a novel method for bone modeling and remodeling [39]. All of these results indicate the possibility that exosomes derived from MSCs play significant roles in intercellular communication between different types of bone cells. 

#### 3.2.2. Immune Cells

Another source from which exosomes can be isolated is immune cells. Exosomes derived from immune cells have immunomodulatory and therapeutic properties, and are crucial for the treatment of cancer. Immune cell-derived exosomes have attracted a lot of interest as a drug delivery platform for effective anticancer therapy, due to their outstanding biocompatibility, low immunogenicity, and high loading capacity [40]. Some researchers have found that the nanovesicles cultured with primary immune cells contain immunoglobulin, which can be used for immunotherapy [41]. Among the various immune cells, neutrophils can respond to inflammation or infection immediately, and are considered to have a dual involvement in both inflammation and malignancy [42]. According to a previous study [43], neutrophil-derived exosomes could eliminate the potential risk of neutrophil phenotype switching from N1 anti-tumoral to N2 pro-tumoral during tumor progression. Xiong et al. [44] have pointed out that M2 macrophage-derived exosomes enriched in miR-5106 could target the salt-inducible-kinase2 (SIK2) and salt-inducible-kinase3 (SIK3) genes to promote the osteogenic differentiation of BMSCs and expedite the healing of fractures. Compared with M2-exosomes, the expression of miR-5106 in M1-exosomes is significantly reduced, suggesting that M1-exosomes may have a stronger osteogenic effect. Numerous studies have demonstrated the value of exosomes derived from various immune cells for therapeutic purposes, as well as the possibility of developing vaccines based on these exosomes. 

#### 3.2.3. Tumor Cells

Exosomes derived from tumor cells are particularly crucial in immune regulation, mediating the cross-talk between malignancies and their intended targets to promote metastasis [45]. Han et al. [46] have demonstrated that it is possible to alter the tumor immune microenvironment to increase tumorigenesis, invasion, angiogenesis, and metastatic dissemination. Through the presentation of tumor antigens by exosomes to T-cells by MHC-I molecules on their surface, an immune response against the tumor is triggered. Exosomes are crucial parts of these interactions, according to previous studies [47], which have shown that the recruitment and migration of immune cells to the tumor microenvironment (TME) are regulated by dynamic signals [48]. For instance, exosomes from tumor cells are possible targets for anti-angiogenic therapy, as they are transported to endothelial cells by endocytosis to cause angiogenesis [49]. Tumor cell-derived exosomes facilitate the initial communication between the primary tumor and metastatic sites [50]. Tumor cells in many sites, including those in the prostate, lung, and breast, are prone to bone metastases and have substantial cross-talk with osteocytes in the bone microenvironment [51]. Amphiregulin (AREG) in multiple myeloma-derived exosomes results in the activation of epidermal growth factor receptor (EGFR) ligand in pre-osteoclasts and is involved in multiple myeloma-induced osteoclastogenesis [52]. This demonstrates that exosomes derived from tumor cells play a significant regulatory function in the metastatic process, and are implicated in pathways related to tumor proliferation or inhibition, bringing new research avenues for the development of oncological drugs.

**Figure 2:**
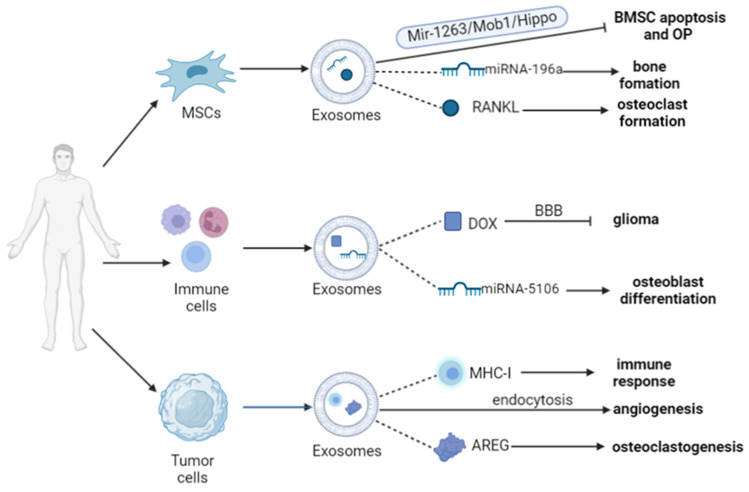
The roles of exosomes from different cells as drug delivery carriers. Exosomes from different cells release a variety of bioactive molecules that are involved in a wide range of diseases.

The experiments mentioned above demonstrate the unquestionable potential of exosomes as therapeutic drug carriers. However, additional research is needed to fully comprehend how exosomes from various cells can be used in DDS (Figure 2). 

### 3.3. Cargo of Exosomes

Various bioactive molecules, including nucleic acids, proteins, lipids, and other small-molecule metabolites, can be carried by exosomes. These may be useful for clinical diagnosis, as they can indicate how blast cells behave and the body’s metabolic state under various disease circumstances.

#### 3.3.1. Nucleic Acid

Exosomes can transport different kinds of nucleic acids. In this review, we primarily introduce recent studies on microRNA (miRNA), long non-coding RNA (lncRNA), and circular RNA (circRNA) in relation to bone metabolism. Exosomes can transfer their cargo to target cells and regulate their growth and differentiation, as was previously described. For example, brown adipose-derived exosomes carrying specific miRNAs can influence lipid metabolism by controlling other organs [53]. It has been found [54] that reducing miR-221-3p in exosomes can significantly inhibit chondrocyte proliferation and migration in vitro. By blocking Runx2 and increasing YAP1-mediated MT1DP, exosomes produced from osteoclasts that contain miR-23a-5p may effectively limit osteogenic differentiation [55]. Exosomal miR-141-3p from MDA-PCa-2b cells also promotes osteoblast activity and regulates the bone metastasis microenvironment, which is crucial for the formation of bone metastases and osteogenesis damage in prostate cancer [56]. lncRNAs are involved in the mechanism whereby exosomes regulate bone metabolism. Studies have shown [57] that, in osteoporotic mice, the exosomal lncRNA MALAT secreted by BMSCs can increase osteoblast activity. Furthermore, the exosomal lncRNA RUNX2-AS1, which is released by multiple myeloma cells, can prevent MSCs from differentiating [58]. Accumulating evidence has indicated that circRNAs play multiple functions in the osteogenesis of BMSCs, proving to be a useful target for the therapy of bone diseases. By interacting with miR-210 and miR-335, CircRNA-0127781 may be one of the key regulators of osteoblast differentiation [59]. CircRNA-33287 has been shown to block mir-214-3p, thus enhancing the osteogenic process and activating the establishment of ectopic bone [60]. CircRNA has a circular structure, which is more stable than RNA and difficult to degrade. Although there are currently few studies on how exosomal circRNAs regulate osteogenic differentiation, this structure holds great promise for future research in exosome engineering. 

#### 3.3.2. Proteins

Apart from delivering different types of nucleic acids, exosomes can also deliver macromolecules, such as proteins. In the study of Liu et al. [61], exosomes have been found to cross the blood–brain barrier (BBB) and specifically target neurons with enkephalins. In addition to protecting the brain against ischemia-reperfusion injury and reducing the chance of negative effects, this also ensures that adequate enkephalin can reach the brain. It has been reported that exosomes containing the anti-inflammatory protein catalase were successfully administered across the BBB to treat Parkinson’s disease [62]. Exosomes carrying the survivin protein, which is a crucial molecule for the detection of cancer, participate in the induction of apoptosis in the TME [63]. Bourdonnay et al. [64] have found that alveolar macrophages (Ams) release exosomes carrying the SOCS1 protein, which alveolar epithelial cells (AECs) may pick up and, thus, limit STAT1 activation and lower inflammatory signals both in vitro and in vivo. As they build up in the bone marrow, antagomir-188—which encourages osteogenic differentiation, inhibits adipogenesis differentiation, and prevents age-related bone loss—is released by CXCR4+ exosome–liposome hybrid NPs [65]. Additionally, research has shown that the PD-L1 antibody-armed exosome vaccination surface can simultaneously prevent PD-1/PD-L1 binding and activate T-cells that are specific to an antigen, in order to treat tumors in a synergistic manner [66]. 

#### 3.3.3. Other Molecules

At present, exosomes can already deliver a number of chemotherapeutic drugs with greater stability and targeting to target cells. Doxorubicin may be effectively absorbed into breast cancer cells when it is enclosed by exosomes, as has been demonstrated by Tian et al. [67], which considerably improves the drug’s therapeutic impact. The therapeutic efficacy of exosomes with paclitaxel formulation against pulmonary metastases has been demonstrated in a Lewis lung carcinoma mouse model [68], revealing the potential of the exosomes as a drug delivery system for the delivery of various chemotherapeutic agents to treatment-resistant cancers. In order to improve the anti-shock function of mice given lipopolysaccharide, Sun et al. [69] have discovered that combining curcumin with exosomes might dramatically increase the anti-inflammatory action of curcumin by enhancing the distribution of curcumin to specific macrophages. Stefano et al. have found that the exosomes possess the ability to carry drugs such as cisplatin [70] and acridine orange [71] into the human body, and can play a key role in the process of tumor metastasis [72]. 

In summary, exosomes are endogenous carriers of active molecules (e.g., nucleic acids, proteins, cytokines, and drugs), which can communicate with other cells and transfer information to particular target cells [73]. Exosome production by cells is typically elevated in many pathologies [74] and, due to the specificity of their cellular origin, exosomes in the blood can even act as biological markers of diseases.

## 4. Role of Exosomes as Drug Delivery Systems in Bone-Related Diseases

As mentioned above, the complex membrane composition of exosomes enables them to have high biocompatibility and stability when they function as drug delivery systems. In addition, exosomes have low immunogenicity and strong osmotic pressure in the body [75]. While delivering drugs accurately, they will not activate the body’s innate or acquired immune system and, so, the drug discharge reaction after entering cells is significantly reduced. Combining the above advantages, exosomes have the ability to pass through the thick tissue barrier of the body to reach the target, transfer the molecules onto the membrane surface or into the cavity to carry out intercellular signal transmission and, finally, achieve the purpose of regulating the physiological function of the target cell [76]. Therefore, exosomes can be widely used as natural biological carriers in bone-related diseases.

### 4.1. Role of Exosomes as a Drug Delivery System for Osteoporosis

Osteoporosis is a systemic metabolic disease in which the dynamic balance of bone reconstruction is disrupted due to abnormal bone metabolism in vivo, resulting in altered bone microarchitecture, decreased bone density and quality, increased bone fragility, and fracture susceptibility. In recent research related to DDS, an increasing number of miRNAs, lncRNAs, and other non-coding RNAs (ncRNAs) have been found to play a crucial part in regulating mRNA translation, gene expression, and other processes through signal transmission [77]. ncRNAs have been found to be widely involved in the development and progression of osteoporosis [76,78,79]. With the discovery of ncRNAs in exosomes, the study of the mechanisms by which exosomal ncRNAs regulate bone remodeling and the role of exosomal ncRNAs in the prevention and treatment of osteoporosis is becoming valuable [80].

In the bone microenvironment, miR-27a-3p, miR-150-3p, and miR-196b-5p secreted by BMSCs promoted the expression of Runx2, Osterix, ALP, and osteocalcin, while knockdown of miR-27a-3p and miR-196b-5p increased the expression of osteoclast markers, leading to bone resorption [81,82]. Some scholars have found that BMSC-Exos carrying miR-935 could reduce the signal transducer and activator of transcription 1 (STAT1) level in osteoblasts of ovariectomized (OVX) rats, where the reduction in STAT1 promoted ALP activity and mineralization deposition in osteoblasts. In vivo studies have shown that silencing miR-935 significantly reduced bone mineral density (BMD), trabecular volume (BV/TV), trabecular number (Tb. N), trabecular thickness (Tb. Th), and increased the characterization of femoral trabecular separation (Tb. SP) [83]. Furthermore, lncRNAs also have a regulatory effect on osteogenic differentiation. Knockdown of lncRNA TCONS_00072128 significantly decreased the expression of cysteine proteases 8 (caspase-8) and osteogenic differentiation of BMSCs. In contrast, lncRNA TCONS_00072128 over-expression upgraded the expression of caspase-8 and osteogenic differentiation of BMSCs [84].

Signaling pathways are integral in the action of exosomes secreted by MSCs as vehicles for drug delivery to treat osteoporosis [85]. Researchers have discovered that BMSC-Exos activates the Wnt/β-catenin pathway to promote osteogenic differentiation, due to its being enriched in miR-196a, and delivers it to HFOB1.19 cells to inhibit Dickkopf-1 (Dkk1), a negative regulator of the Wnt/β-catenin pathway [86]. Experiments in mouse models have confirmed that the miR-19b carried by BMSC-Exos could inhibit WWP1 or Smurf2 and increase KLF5 expression through the Wnt/β-catenin signaling pathway, and that activation of the KLF5/β-catenin signaling pathway up-regulates the expression of osteogenic factors (Col I, ALP, and Runx2), and promotes the mineralization process of osteoblasts, thus stimulating osteogenesis [87]. The miR-136-5p carried by BMSC-Exos activates the Wnt/β-catenin pathway by targeting LRP4, and in vitro experiments have found that the delivery of BMSC-Exos-derived miR-136-5p to MC3T3-E1 could promote the proliferation and osteogenic differentiation of MC3T3-E1, thereby promoting osteogenesis [88].

Other tissue-derived stem cells have further impacts, in addition to the modulatory effect of exosomes secreted by BMSCs on osteoporosis. ADMSCs secrete phenotypically characterized miR-146a-containing exosomes, due to the over-expression of miR-146a in diabetic osteoporosis. Exosomes containing miR-146a can reduce the production of inflammatory cytokines in high-glucose-treated osteoclasts and inhibit bone resorption, which had the most effective effect on bone loss in diabetic osteoporotic rats [89]. It has been shown that miR-5106 is highly enriched in macrophage 2-derived exosomes (M2D-Exos), and can be transferred to BMSCs, targeting SIK2 and SIK3 genes to promote osteoblast differentiation [44]. Exosomes derived from human-induced pluripotent stem cells (iPSCs-Exos) effectively stimulated osteogenic differentiation and proliferation in OVX rats, and the effect increased with increasing exosome concentration [90]. 

Angiogenesis and osteogenesis are closely coupled in the process of bone remodeling. BMSC-Exos carrying miR-29a have been shown to regulate osteogenesis and angiogenesis concurrently, and a large increase in bone mass can be induced through the regulation of miR-29a on angiogenesis by targeting VASH1 [91]. In addition, by acting as an miR-106a sponge, lncRNA H19 transported by exosomes is capable of inhibiting miR-106a. While competitively binding to miR-106a, lncRNA H19 enhances Angpt1-Tie2 signal transduction, increases NO production, and promotes new blood vessel formation and bone mineralization [92]. Another study has indicated that UMSC-Exos does not directly regulate the expression of osteogenic-related genes but, rather, regulates HIF-1α to accelerate bone regeneration by mediating the stimulation of VEGF expression and angiogenic capacity [93].

**Figure 3:**
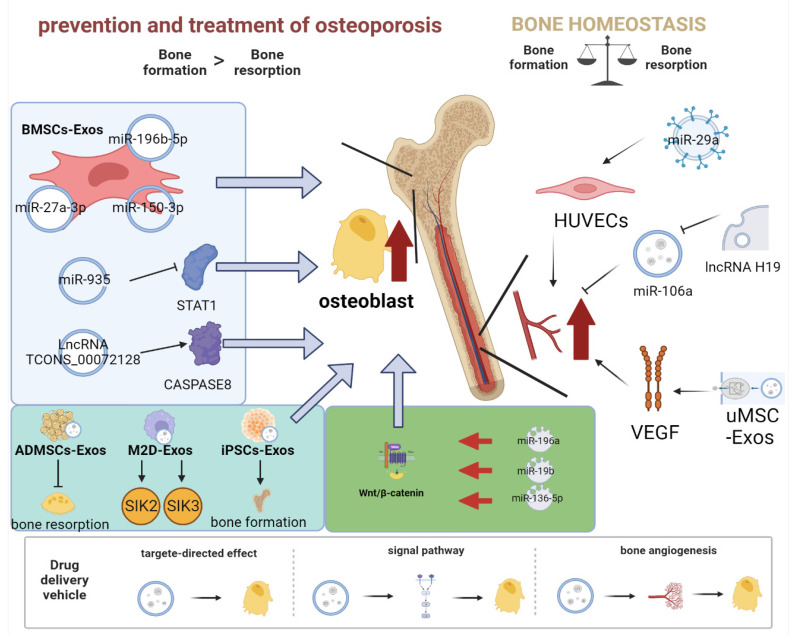
Exosomes act as drug delivery systems in osteoporosis. Exosomes can carry bioactive molecules that act directly on osteoblasts and promote osteoblast proliferation through signaling pathways, while bone angiogenesis also provides a way to prevent osteoporosis.

In summary, exosomes derived from MSCs of different origins have presented unprecedented potential applications in the treatment and intervention of osteoporosis. Secreted exosomes can target receptor cells as part of a drug delivery system or modulate a range of signaling pathways affecting bone formation and bone angiogenesis, thus offering a fresh approach to treating osteoporosis (Figure 3). 

### 4.2. Role of Exosomes as a Drug Delivery System for Fracture

Fractures are the most common physical trauma in humans [94]. It has been estimated that 5–10% of fractures may result in non-union of the fracture, thus adding a longer return to function for patients, causing them a greater financial burden and leading to a waste of medical resources [95]. Fracture healing is a complex physiological process involving the coordinated involvement of multiple cells. A moderate inflammatory response precedes fracture healing, but overactive and persistent inflammation can lead to tissue damage and delay the fracture healing process, while adequate fracture healing can be promoted by suppressing locally over-active immune cells at the fracture site. PD-L1 from exosomes of human umbilical vein endothelial cells (HUVECs) encapsulated in hydrogels promoted tissue formation and healing of fractures in a mouse model of early inflammatory hyperactivity [96].

Recent studies have shown that exosomes play significant roles in the regulation of fracture repair [97]. Based on the continued development of regenerative medicine and bone tissue engineering approaches, MSCs are becoming increasingly promising and attractive as a medium for fracture repair. Recent evidence has suggested that MSC-derived exosomes can provide an effective therapeutic strategy for bone repair [98]. 

Numerous studies have shown that exosomes are a new factor of paracrine signaling in BMSCs, playing an important role in tissue repair processes [37]. BMSCs-Exos significantly enhanced the osteogenic differentiation of BMSCs in vitro. Intravenous administration of the MSC–Exo–Aptamer complex in mice has been found to enhance bone mass in OVX rats and accelerate bone healing in a mouse model of femur fracture [99]. Furthermore, in a rat model of non-union, noggin and LDN193189—inhibitors of BMP-2—were used and BMSCs-Exos were found to significantly promote osteogenesis, angiogenesis, and bone healing processes in the rat femur [100]. In a study of BMSC-Exos for the treatment of obesity-induced fractures, miR-467 was found to function as a regulatory molecule for lncRNA H19 and Hoxa10 and to improve fracture healing [101]. 

The paracrine effect of BMSCs can also be enhanced under hypoxic conditions. It has been demonstrated that BMSCs-Exos under hypoxic (sub-Exos) conditions promote angiogenesis, proliferation, and migration more strongly than exosomes, and that a reduction in the amount of hypoxia-inducible factor 1 (HIF-1α) leads to a substantial decrease in miR-126 in BMSCs and exosomes, thereby eliminating the role of sub-Exos, thus also demonstrating that sub-Exos promotes fracture healing through miR-126 [97].

It has been found that UMSCs not only participate in fracture repair through signaling pathways in addition to BMSCs-Exos, but also promote angiogenesis for fracture healing. By injecting UMSC-Exos into the fracture sites of Sprague Dawley (SD) rats, we have previously observed better bone apposition and continuity of cortical bone; increased protein expression levels of Wnt3a and β-catenin in the Wnt signaling pathway; and significantly higher expression levels of COL-1, OPN, and RUNX2. All of these results demonstrate that UMSC-Exos may be involved in fracture repair in rats through the Wnt signaling pathway [102]. In a rat femur fracture model, it has also been found that UMSC-Exos significantly promoted angiogenesis and bone healing [93]. Additionally, ADSC-Exos function as an immunomodulator in the treatment of bone-related diseases. ADSC-Exos-derived miR-451a has been shown to inhibit macrophage migration inhibitory factor (MIF), which can promote the M1 to M2 polarization of macrophages, inhibit inflammation, and simultaneously accelerate bone healing [103]. 

In addition, experts have developed a cocktail therapy that simultaneously regulates the balance of M1/M2 macrophages and osteoblasts/osteoclasts, consisting of a natural polymer hyaluronic acid hydrogel, an engineered endothelial cell-derived exosome (EC-ExosmiR-26a-5p), and the IRE-1α inhibitor APY29; their results also showed that the cocktail therapy acts to promote fracture repair [104].

**Figure 4:**
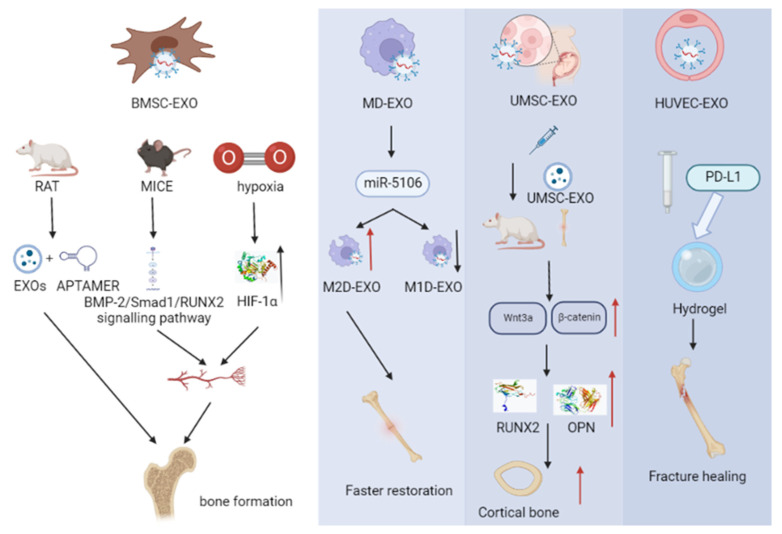
Exosomes act as drug delivery systems in fractures. Exosomes secreted from different stem cells can lead to osteogenic differentiation and promote bone healing.

In summary, as a natural biological carrier for drug delivery, exosomes not only can improve fracture healing by promoting osteogenic differentiation through paracrine activation, but also enhance bone healing through signaling pathways and bone angiogenesis. The exosomes secreted by stem cells in a hypoxic environment have a more obvious effect on fracture recovery, which also provides a new idea for promoting fracture healing in the future (Figure 4). 

### 4.3. Role of Exosomes as Drug Delivery Systems for Osteoarthritis

Osteoarthritis is a prevalent chronic joint disease, characterized by secondary bone hyperplasia and degenerative changes in the articular cartilage. An aging population and more patients with obesity are becoming more prominent with socio-economic development, leading to a gradual rise in the incidence of osteoarthritis [105]. The habits and lifestyles of patients are greatly impacted by osteoarthritis, which significantly interferes with their daily lives. Non-steroidal anti-inflammatory drugs (NSAIDs) are now widely used in medical settings; however, their effects are primarily focused on pain relief, which lessens patient discomfort, rather than cartilage regeneration, eventually resulting in further deterioration of the joints [106]. With the continuous development of regenerative medicine, a large number of studies have found that stem cells can be used to repair tissues or organs, especially stem cell-derived exosomes [106].

**Figure 5:**
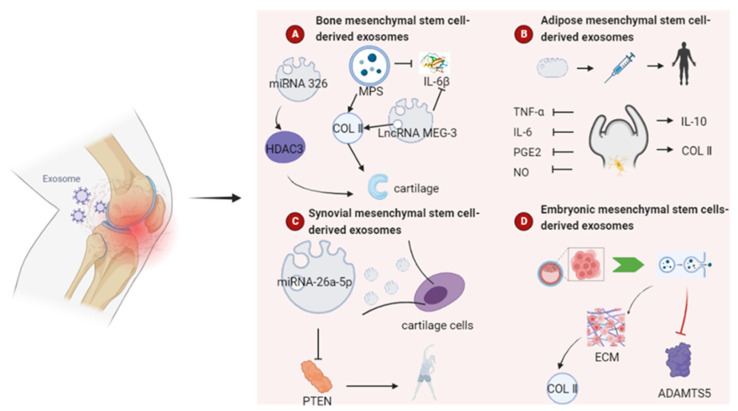
Role of exosomes as drug delivery systems for osteoarthritis. Exosomes derived from different mesenchymal stem cells can be used as DDS to treat osteoarthritis.

Exosomes, as key vehicles for intercellular signal transmission, provide a more advantageous way to evaluate the pathological cells or tissue morphology in osteoarthritis [107]. In addition to promoting angiogenesis, bone remodeling, and chondrocyte proliferation and migration, exosomes can inhibit osteoarthritic chondrocyte apoptosis and reduce cartilage inflammation [107]. Therefore, exosomes derived from BMSCs, adipose mesenchymal stem cells (AMSCs), synovial mesenchymal stem cells (SMSCs), human embryonic stem cells (HESCs), and other types of MSCs can be used as DDS to treat osteoarthritis [108] (Figure 5).

#### 4.3.1. Bone Mesenchymal Stem Cell-Derived Exosomes

Using a rat model of osteoarthritis, researchers have found that BMSCs and BMSC-Exos both improved joint cavity and articular cartilage of osteoarthritis rats, alleviated articular cartilage and joint site damages, and restored trabecular bone volume fraction and trabecular bone number in osteoarthritis rats. Additionally, in vitro studies have revealed that BMSC-Exos carrying lncRNA MEG-3 potentially prevent interleukin-1 (IL-1)-induced senescence and apoptosis, while enhancing the production of type II collagen to retain the chondrocyte phenotype [109]. BMSC-Exos have also been found, by He et al. [110], to efficiently stimulate cartilage regeneration and extracellular matrix formation while reducing knee joint discomfort in osteoarthritis rats. Their study also indicated that BMSC-Exos carrying lncRNA MEG-3 has an anti-osteoarthritis targeting effect. Meanwhile, another researcher has discovered that the inhibitory effect of IL-1β on chondrocyte proliferation and migration was dramatically reduced by exosome administration. Exosome pre-treatment considerably reduced the effects of IL-1β on the down-regulation of COL2A1 and ACAN, as well as the up-regulation of matrix metallopeptidase 13 (MMP13) and a disintegrin and metalloproteinase with thrombospondin motifs 5 (ADAMTS5) [110].

In addition to BMSCs-Exos acting in osteoarthritis, the exosomes themselves can act as drug delivery vehicles to deliver miRNAs to chondrocytes and improve the symptoms of osteoarthritis. Exosomal miR-326 generated from BMSCs can target histone deacetylase 3 (HDAC3) and STAT1//NF-kB p65 to prevent chondrocyte and cartilage from pyroptosizing, transfer mir-326 to chondrocytes and cartilage, and reduce the symptoms of osteoarthritis [111]. Studies have suggested that E2F2 is an important member of the E2F family of transcription factors, which are involved in cell proliferation, differentiation, and apoptosis processes [112]. miR-125a-5p has the ability to accelerate chondrocyte migration, and exosomal miR-125a-5p produced by BMSCs enhances chondrocyte migration while inhibiting cartilage degradation by targeting E2F in traumatic osteoarthritis cartilage tissue [113].

Some researchers have also found that TGF-β is an effective inducer of chondrocyte anabolism, and BMSCs-Exos treated with TGFβ3 significantly up-regulated the expression of chondrogenic genes (COL1, COL2B, and ACAN) in osteoarthritis and down-regulated inflammatory marker genes (MMP-13, ADAMTS5, and iNOS), which protected chondrocytes from apoptosis and inhibited macrophage activation; therefore, they presented cartilage-protective and anti-inflammatory properties, preventing the development of osteoarthritis in mice [114]. It is thus reasonable to believe that exosomes derived from BMSCs have promising potential for the development of osteoarthritis treatment through drug delivery.

#### 4.3.2. Adipose Mesenchymal Stem Cell-Derived Exosomes

Regarding the use of regenerative therapies, it has been demonstrated that AMSCs are able to heal damaged cartilage in osteoarthritis due to their chondrogenic potential, capacity for self-renewal, and immunomodulatory properties [115,116]. Therefore, they are considered to be a preferable cell source for osteoarthritis treatment.

Exosomes have been shown to act as a mediator for the paracrine function of AMSCs, which decreased the generation of inflammatory mediators, by Tofino Vian et al. [117]. In a 6-month experiment [118], in which 12 patients (MSC group) were given AMSCs and compared with 12 patients (control group) whose knees were injected with normal saline, the changes in cartilage defects after injection were evaluated by magnetic resonance imaging (MRI). The MRI results indicated that cartilage defects in the MSC group had no significant changes at 6 months, while cartilage defects in the control group increased. For outpatient patients with knee osteoarthritis, intra-articular injections of autologous AMSCs provided acceptable functional improvement and pain relief. 

Furthermore, AMSC-Exos boost anti-inflammatory cytokines while lowering the production of inflammatory mediators, among which annexin A1—a component of exosomes—may participate in the anti-inflammatory process. In the chondrocytes in osteoarthritis, exosomes have been shown to reduce inflammatory mediators such as tumor necrosis factor-α (TNF-α), IL-6, prostaglandin E2 (PGE2), and NO [117]. The down-regulation of cyclooxygenase 2 (COX-2) and microsomal prostaglandin E synthase 1 (mPGES-1) can reduce the production of PGE2, and the effect on NO may be contingent on a decrease in the expression of inducible NO synthase. Treatment of osteoarthritis with extracellular vesicles also reduced MMP activity and the expression of MMP-13, while the production of the anti-inflammatory cytokine IL-10 and the expression of col II was markedly increased. These results suggest that exosomes from AMSCs have prospective treatment potential for osteoarthritis. 

#### 4.3.3. Synovial Mesenchymal Stem Cell-Derived Exosomes

The synovium is an essential part of the synovial joint, which contributes significantly to the maintenance of articular cartilage homeostasis. Several studies have shown that the number and composition of synovial interstitial-derived exosomes may change in joint diseases, and that exosomes derived from SMSCs may also be used in the treatment of osteoarthritis.

Synovial mesenchymal stem cell-derived exosomes (SMSC-Exos) have been shown [119] to significantly promote cartilage regeneration and inhibit the development of osteoarthritis. SMSC-Exos carrying miR-26a-5p have been shown to inhibit PTEN, thereby suppressing cell apoptosis, inflammation, and improving cartilage damage in osteoarthritis [120]. It also activates the Yes-associated protein by dephosphorylating and reducing extracellular matrix secretion, as well as inducing articular chondrocyte proliferation and migration through Wnt5a and Wnt5b signaling [121].

In a different set of studies, induced pluripotent stem cell-derived-exosomes MSCs (iMSCs-Exos) and SMSC-Exos were injected into the joints in a collagenase-induced osteoarthritis model. The results showed that both iMSC-Exos and SMSC-Exos stimulated the migration and proliferation of chondrocytes, where iMSC-Exos played a stronger role [119].

#### 4.3.4. Other Sources of Mesenchymal Stem Cells

The exosomes secreted from embryonic mesenchymal stem cells have been shown to attenuate the development of osteoarthritis by regulating the balance between the production of cartilage matrix and degeneration. Embryonic stem cells can enhance the expression level of Col II in the cartilage matrix and reduce the expression of ADAMTS5, providing a new target for the development of osteoarthritis drugs and drug delivery systems [122].

Induced pluripotent stem cells (iPSCs) have certain effects on osteoarthritis, as they have the potential to simulate diseases and can be applied to clinical environments [123], being similar to embryonic stem cells in structure, self-regeneration, and specialization [124].

At the same time, the exosomes targeting chondrocytes have been used as carriers to deliver miR-140 to chondrocytes as a new method to treat osteoarthritis. CAP-Exos, which can successfully wrap miR-140 and enter into and distribute miR-140 in chondrocytes in vitro, are created by combining the chondrocyte affinity peptide (CAP) with the lysosomal associated membrane glycoprotein 2b protein on the surface of exosomes. After intra-articular injection, CAP-Exos remain in the joints, compared to unlabeled exosome vesicles, and internal diffusion is negligible. Additionally, CAP-Exos potentially blocked cartilage breakdown protease, transferred miR-140 to the deep cartilage area through thick medium cartilage, and slowed the course of osteoarthritis in a rat model [125].

### 4.4. Role of Exosomes as Drug Delivery Systems for Osteosarcoma

Osteosarcoma, one of the most common primary bone cancers in adolescents, is basically derived from primitive mesenchymal stem cells. The age distribution of osteosarcoma is bimodal, with the first peak occurring at 15–19 years and the second peak occurring at 75–79 years [126]. Notably, males are more frequently affected than females, in general. 

Exosomes are essential in tumorigenesis, proliferation, metastasis, anti-apoptosis, immune evasion, and chemotherapy resistance in osteosarcoma [127]. Mesenchymal stem cells communicate with other cells through pathways including EVs. Due to their unique properties, MSCs-derived exosomes (MSC-Exos) can be altered and processed into effective biological carriers, loaded with drugs, transfected with anticancer genes, and employed for targeted therapy of osteosarcoma [128]. Experiments have further demonstrated that the targeting ability of exosomes is due to the chemotaxis of MSC-Exos to osteosarcoma cells by the SDF1-CXCR4 axis [129]. It has been reported that exosomes isolated from MSCs can be used as drug nanocarriers to load the chemotherapeutic drug doxorubicin (DOX), and that the exosome-loaded doxorubicin (Exo-DOX) could be taken up by MG63 cells, thus inducing cell death. Therefore, the modified MSC-Exos can serve as an excellent nanocarrier to target the tumor site and have a better anti-cancer impact [130]. 

In the area of in vivo drug administration, MSC-Exos offer significant research potential and a wide range of application possibilities, which can lead to novel research techniques and concepts for cancer therapy. However, relevant research is still at the pre-clinical stage, and more fundamental research is required to elucidate the related molecular mechanisms [131]. 

Exosomes have been shown to regulate the development of osteosarcoma as part of drug delivery, and can positively affect osteosarcoma through signaling pathways such as Wnt/β-catenin, ERK1/2, and JAK2/STAT3. Sha et al. [132] have discovered that Hic-5 could regulate the Wnt/β-catenin signaling pathway through exosomes [133], thereby inhibiting the proliferation of osteosarcoma cells. Exosomal miR-208a derived from the BMSC has been shown to negatively target programmed cell death protein 4 (PDCD4) in order to activate the ERK1/2 signaling pathway, thus promoting the proliferation, migration, and invasion of osteosarcoma cells [127]. It has been reported [134] that lymphocyte cytoplasmic protein 1 (LCP1) in BMSC-Exos promotes the progression of osteosarcoma through the JAK2/STAT3 pathway, suggesting that targeting LCP1 may provide a means to treat osteosarcoma.

Some miRNAs are widely involved in the regulation of osteosarcoma through exosome-based drug delivery systems. By affecting apoptosis-related genes and signaling pathways, including the mitochondrial apoptosis route, the death receptor pathway, and the endoplasmic reticulum system, miRNAs can control the apoptosis of osteosarcoma cells. Exosomes can be viewed as a useful technique for the early diagnosis of osteosarcoma [135], as these aberrant miRNAs can be stored and distributed through exosomes. During the progression of osteosarcoma, miRNAs can be delivered to osteosarcoma cells by exosomes derived from non-tumor cells such as MSCs and AMSCs. It has been observed [136] that miR-206 may be carried by and transported to osteosarcoma cells by BMSC-Exos. Results from both in vitro and in vivo experiments have indicated that BMSC-Exos carrying miR-206 may prevent osteosarcoma cells from proliferating, migrating, and invading, as well as inducing apoptosis in osteosarcoma cells. BMSC-Exos containing miR-206 inhibited the progression of osteosarcoma by targeting transformer 2β (TRA2B) [137]. It has also been shown that synthetic miR-143 introduced into cells was encapsulated and released by exosomes, and miR-143 formed by secreted exosomes was transferred to osteosarcoma cells and prevented the 143B osteosarcoma cell line from migrating.

Moreover, CASC15 is a novel tumor-related lncRNA, the abnormal expression of which has been reported in a variety of tumors, demonstrating that CASC15 is up-regulated in plasma exosomes of patients with osteosarcoma, while CASC15 knockdown can inhibit the progression of osteosarcoma by targeting the miR-338-3p/RAB14 axis. As a possible new therapeutic target for the treatment of osteosarcoma, the exosomal lncRNA LIPR-AS1, which is produced by macrophages, can increase osteosarcoma cell proliferation and invasion while decreasing cell apoptosis [138]. 

The engineering of AMSCs to secrete miR-101-enriched EVs has been shown to provide effective results. These EVs exert inhibitory effects on cell invasion and migration in vitro once they have been taken up by osteosarcoma cells. Experimental data have supported the function of miR-101 as an osteosarcoma tumor suppressor by down-regulating B-cell lymphoma 6 (BCL6). AMSC-derived miR-101-enriched EVs represent a potential innovative therapy and promising circulating biomarker for metastatic osteosarcoma [139]. These results provide a novel method for the therapy of osteosarcoma, in which exosomes are secreted by other cells and contain specific substances to slow the growth of the disease and exert therapeutic effects on osteosarcoma cells.

Overall, the existing evidence indicates that exosomes, as cellular communication molecules, are very promising for the targeted therapy of osteosarcoma. To date, numerous studies have focused on the role of exosomes derived from MSCs in this context. As exosomes can be derived from a variety of cells, future studies should continue to expand research regarding the source, effectiveness, and specific mechanisms of various exosomes. 

## 5. Conclusions and Future Prospect

Within the last decade, exosomes have been extensively studied as key mediators of intercellular communication. The contents of exosomes, such as lipids, proteins, and genetic material, have shown great potential for the diagnosis and treatment of bone-related diseases. Studies have indicated that exosomes may have advantages as drug carriers, in terms of biocompatibility, stability, and intrinsic targeting, as they have been found to promote angiogenesis, osteoblast differentiation, and bone tissue regeneration through a variety of signaling pathways; however, their use in drug delivery systems is still in its infancy. The majority of research on exosomes is still focused on cellular and animal models and lacks clinical applications. Most experiments with exosomes in animal models are still conducted using tail vein injection; however, the ability of this method to effectively deliver exosomes to the lesion remains to be investigated. Due to the limitations of the production process, the extraction efficiency of exosomes is low. Additionally, some technical, functional, and safety issues regarding exosome-based drug formulations remain unresolved, and there is still a long way to go from laboratory studies to clinical applications.

Nevertheless, it is good to know that, with the rapid expansion of research on exosomes in bone homeostasis, we now have a better understanding of the pathogenesis and treatment of exosomes in bone-related diseases. Exosomal drug delivery methods are anticipated to have greater therapeutic results for a variety of ailments as innovation advances. Likewise, it is anticipated that the miRNA, lncRNA, and proteins carried by exosomes generated from cells related to bone may serve as possible bone markers to assess and identify disorders connected to bone. In addition, in view of the current threat of COVID-19, some people have advocated for the use of extracellular vesicles to treat COVID-19 and even support stem cell therapy and exosome products derived from the umbilical cord, amniotic fluid, and other sources, which they believe can both prevent and treat COVID-19. The wide range of actions of exosomes must be further investigated in the future, in order to provide further possibilities for study in the disciplines of biomedicine and biomaterials, given the significant potential of exosomes for drug delivery in drug therapy.

## Data Availability

Not applicable.
